# A phase 1 dose-escalating study of pegylated recombinant human arginase 1 (Peg-rhArg1) in patients with advanced hepatocellular carcinoma

**DOI:** 10.1007/s10637-012-9807-9

**Published:** 2012-03-17

**Authors:** Thomas Yau, P. N. Cheng, Pierre Chan, William Chan, Li Chen, Jimmy Yuen, Roberta Pang, S. T. Fan, Ronnie T. Poon

**Affiliations:** 1 Department of Medicine, The University of Hong Kong, Queen Mary Hospital, Pokfulam, Hong Kong, China; 2 Department of Surgery, The University of Hong Kong, Queen Mary Hospital, 102 Pokfulam Road, Pokfulam, Hong Kong, China; 3 Centre for Cancer Research, The University of Hong Kong, Queen Mary Hospital, Pokfulam, Hong Kong, China; 4 State Key Laboratory of Liver Diseases, The University of Hong Kong, Queen Mary Hospital, Pokfulam, Hong Kong, China; 5 Department of Medicine, Ruttonjee Hospital, 266 Queen’s Road East, Wan Chai, Hong Kong; 6 Bio-Cancer Treatment International Limited, Rm 511-513, 5/F, Bio-Informatics Centre, 2 Science Park West Avenue, Hong Kong Science Park, Shatin, NT Hong Kong; 7 Hong Kong Sanatorium & Hospital, 2 Village Road, Happy Valley, Hong Kong

**Keywords:** Arginine, Advanced HCC, Arginase, Peg-rhArg1

## Abstract

*Background* Hepatocellular carcinoma (HCC) cells are auxotrophic for arginine, depletion of which leads to tumour regression. The current study evaluated safety, pharmacokinetics (PK)/ pharmacodynamics (PD) parameters, and potential anti-tumor activity of pegylated recombinant human arginase 1 (peg-rhArg1) in advanced HCC patients. *Methods* Eligibility criteria included advanced HCC with measurable lesions, Child-Pugh A or B, and adequate organ function. Initial single IV bolus was followed by weekly doses of peg-rhArgI escalated from 500 U/kg to 2500 U/kg in a 3 + 3 design. *Results* Fifteen patients were enrolled at weekly doses of 500 U/kg (*n* = 3), 1000 U/kg (*n* = 3), 1600 U/kg (*n* = 3) and 2500 U/kg (*n* = 6). The median age was 57 years (33–74); 87% were hepatitis B carriers and 47% had prior systemic treatment. The most commonly reported drug-related non-haematological adverse events (AEs) were diarrhea (13.3%), abdominal discomfort (6.7%) and nausea (6.7%). No drug-related haematological AEs were seen. Only 1 of the six patients that received 2500U/kg peg-rhArg1 experienced DLT (grade 4 bilirubin elevation) and thus the maximum tolerated dose was 2500 U/kg. PK and PD analysis indicated that peg-rhArg1 was efficacious in inducing arginine depletion in a dose-dependent manner. Adequate arginine depletion dose was achieved in the 1,600–2,500 U/kg range and therefore the optimal biological dose was at 1600 U/kg, which was chosen as the recommended dose. The best response was stable disease for >8 weeks in 26.7% of the enrolled patients. *Conclusion* Peg-rhArg1 has manageable safety profile and preliminary evidence of activity in advanced HCC patients.

## Background

Hepatocellular carcinoma (HCC) is the third most common cause of cancer-related death [1]. While long-term survival of patients with early HCC amenable to surgical treatment has improved in recent years, prognosis of patients with advanced disease remains poor with the median overall survival (OS) usually around 3 months from the time of diagnosis of the advanced disease [2].

HCC is relatively chemo-resistant. Thus far, there are no convincing phase III data to suggest that systemic chemotherapy, whether singly or in combination, improves overall survival in patients with advanced disease [3]. Notably, the use of the new generation of chemotherapy, such as oxaliplatin has recently demonstrated seemingly encouraging results. In particular, the results of the randomised phase 3 trial in comparing the efficacy of oxaliplatin plus 5-Flurourcail and Leucovorin versus doxorubicin (FOLFOX4) as the palliative treatment for advanced HCC was reported in the ASCO meeting 2010 by Quinn et al [4]. The preliminary results showed that FOLFOX resulted in better overall survival and better tolerability than patients received doxorubicin. Thus, the future role of oxaliplatin in the treatment algorithm of advanced HCC patients is being investigated in the ongoing trials in advanced HCC population. Recently, two pivotal phase-III randomized placebo-controlled trials in the West [5] and Asia-Pacific region [6] have shown benefit with single agent sorafenib in patients with advanced HCC. Based on these studies, sorafenib was approved for the management of advanced HCC patients. It is noteworthy that the OS of patients on sorafenib was only 6.5 months, compared with 4.2 months in patients on placebo [6]. Thus, the overall improvement in OS is around 2.3 months. Furthermore, the treatment side effects from sorafenib, such as hand-foot-skin reaction and fatigue should not be lightly dismissed. Therefore, an unmet medical need remains to develop more effective therapeutic agents with significantly less side effects in the treatment of advanced HCC.

Arginine is a semi-essential amino acid involved in the synthesis of a wide range of peptides, proteins, and a myriad of metabolic pathways and cellular events [7]. HCC cells are said to be auxotrophic for arginine since they cannot, like normal somatic cells, recycle arginine from citrulline, making them completely reliant on exogenous arginine for growth. Thus, there are potential benefits in using arginine deprivation therapy in treating HCC. In vitro, arginine depletion strongly induced tumour regression in argininosuccinate synthase (ASS) deficient HCC cell lines [8]. In vivo, xenograft studies confirmed that arginine depletion inhibited tumour growth in murine HCC, which do not express ASS mRNA. However, when it was transfected with an expression plasmid containing ASS cDNA, the transfected cells were much more resistant to arginine depletion [8, 9].

Two major enzyme therapeutics, pegylated arginine deiminase and pegylated human hepatic arginase, are now in various stages of clinical development. Arginine deiminase (ADI) is a *Mycoplasma* secreted enzyme that degrades arginine to citrulline in vivo, releasing ammonia as the other reaction product [10]. It may control growth of ASS deficient or arginine auxotrophic HCC. The pegylated form of ADI is now in phase-II clinical trial in HCC and melanoma and is reported to have moderate disease-stabilizing activity in HCC [11]. Arginase is another arginine-degrading enzyme for use in the treatment of arginine auxotrophic tumours. Unlike ADI, this non-xenogenic and native (human) enzyme hydrolyses arginine to ornithine and urea; the latter is non-toxic and excreted in the urine. The arginase used in this trial was of Good Manufacturing Practice (GMP) grade-- pegylated recombinant human arginase1 (peg-rhArg1), from an *E. coli* expression system. After suitable pegylation to increase its in vivo half-life in plasma from a few hours to 3–4 days, it has been shown to have strong in vitro and in vivo anti-tumour activities in a number of tumour types including HCC [9]. Production of this pegylated recombinant human arginase 1 has been reported elsewhere [12].

The aim of this open-label, phase I dose-escalation study was to evaluate safety, pharmacokinetics (PK)/pharmacodynamics (PD), and potential anti-tumor activity of peg-rhArg1 in patients with advanced HCC.

## Patients and methods

This was a single-center, prospective, open-label, phase 1 dose-escalation study of peg-rhArg1 in subjects with advanced HCC unsuitable for either surgical or loco-regional therapy. The protocol was approved by the local IRB and Ethics Committee with written consents being obtained from the patients before enrollment. The trial was registered in clinicaltrials.gov identifier NCT01092091 and conducted in compliance with ICH guidance of Good Clinical Practice.

### Patient’s eligibility

Advanced HCC patients not suitable for surgery or various loco-regional therapies at the Queen Mary Hospital, Hong Kong, were recruited. HCC was diagnosed either by cyto-histological confirmation or by non-invasive criteria according to the European Association for Study of Liver disease (EASL) criteria: cirrhotic patients with either two coincident imaging techniques demonstrating focal lesion >2 cm with arterial hypervascularization or one imaging technique showing focal lesion >2 cm with arterial hyper-vascularization and associated with alpha fetal protein(AFP) level >400 ng/ml [13]. Staging was by American Joint Committee on Cancer (AJCC) and Barcelona Clinic Liver Cancer (BCLC) score. The eligibility criteria included adult patients 18–75 years old; patients with Child-Pugh class A or B cirrhosis; Karnofsky performance status (KPS) ≥ 80%; expected life expectancy of ≥12 weeks and with adequate organ function: complete blood picture (absolute neutrophil count (ANC) >1.0 × 10^9^/L, platelet count >100 × 10^9^/L) and biochemistry (total bilirubin of ≤2 × upper limit of normal, serum AST and ALT ≤ 5 × upper limit of normal). The disease must be measurable with at least one lesion, which was at least 2 cm in one dimension either on CT or MRI scan. All the enrolled patients had clear progressive disease with their last treatment modalities prior to study entry.

### Dosing scheme, dose limiting toxicity and maximum tolerated dose

Patient enrollment was based on the 3 + 3 paradigm with dose-escalating cohorts as shown in Table 1. The escalation scheme followed the modified Fibonacci scheme commonly used in phase I trials [14]. DLT was defined as any grade 4 toxicities, grade 3 neutropenia or the occurrence of neutropenic sepsis, grade 3 thrombocytopenia or any grade 3 toxicities that did not return to grade1/2 within 3 weeks, except for alopecia. The maximum tolerated dose (MTD) was defined as the highest weekly level of IV bolus peg-rhArg1 at which no more than one of six patients experienced a DLT.

**Table 1:** Patient cohorts and dosing scheme

Cohort	U/kg
One	500
Two	1000
Three	1600
Four	2500

**Fig. 1:**
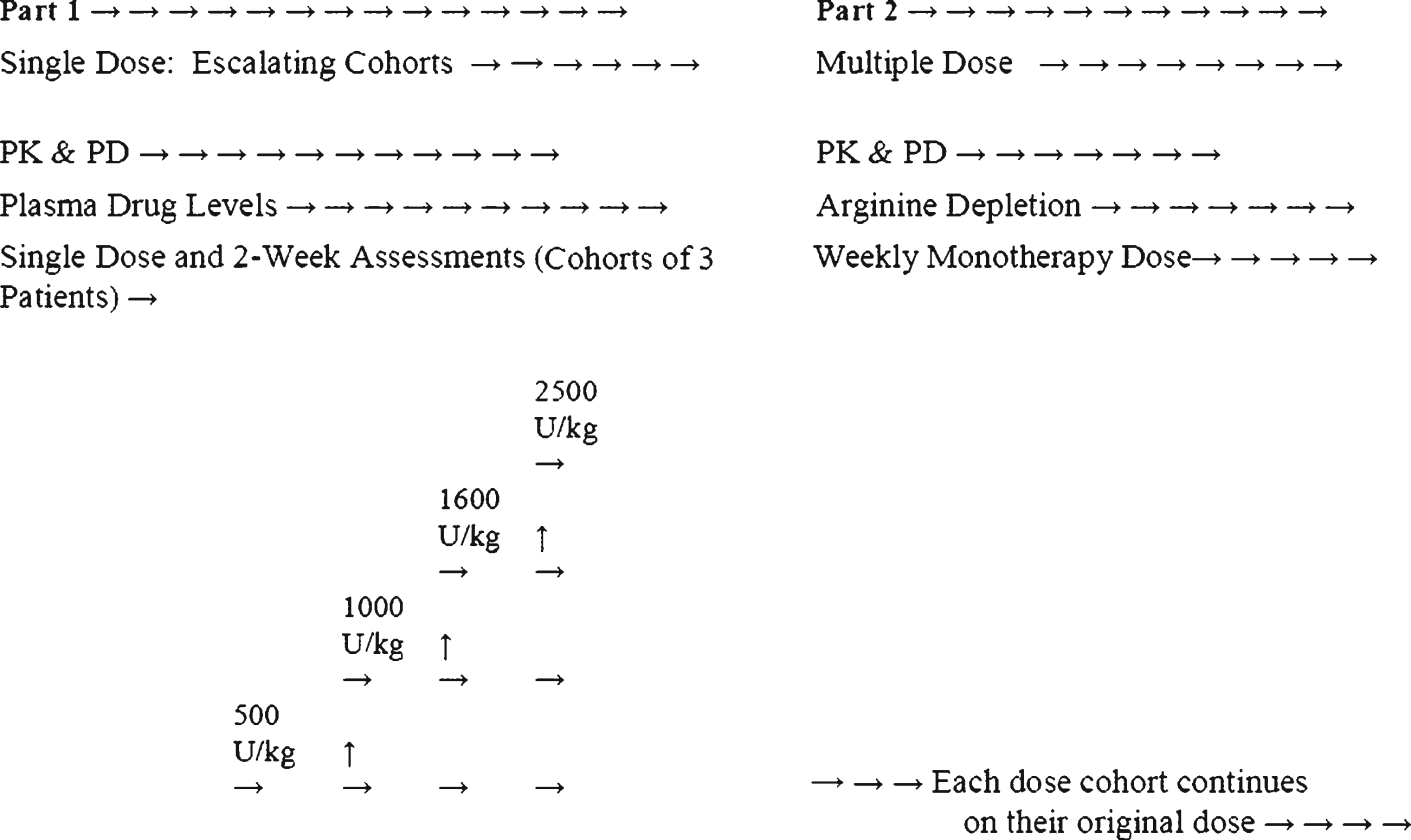
Dose escalating scheme of Peg-rhArg1

Initially, a cohort of 3 advanced HCC patients received a bolus I.V. administration of peg-rhAgr1 started at 500 U/kg. Single dose safety parameters, including hematology and chemistry laboratory profiles, were monitored weekly for 2 weeks. Patients did not demonstrate a dose limiting toxicity ( DLT) following the single dose subsequently received weekly IV bolus of peg-rhArg1 at the same dose level from week 3 (day 15) onwards (Fig. 1).

### Pharmacokinetics(PK) and Pharmacodynamics (PD) measurement

Plasma arginase and corresponding arginine levels were measured following bolus I.V. administration of peg-rhArg1 in groups of 3 patients given a range of peg-rhArgI. After the dose for each patient, blood samples for plasma arginase were drawn at baseline, 2 and 4 h after the single dosing to establish maximum plasma concentration (C_max_) and initial clearance. Additional single time-point samples were drawn on Days 2, 4, 6 and 8 to establish the terminal T_1/2_ and duration of arginine depletion after the first dose. During weekly dosing, baseline (pre-dose) samples and 24 h post dose samples were obtained from weeks 3 to 5; then only one baseline sample from weeks 6 to 11. PK parameters to be calculated based on a non-compartmental model approach include: C_max_, time to maximum observed concentration (T_max,_), minimum observed concentration (C_min_), and area under the curve (AUC_∞_). Arginase levels were assessed using ELISA Kit (BEIJING 4A BIOTECH Co. LTD, in China). The methodology of these tests is per standard ELISA protocol. Arginine level was analyzed by a high-speed amino acid analyzer [15]. Adequate Arginine Depletion (ADD) dose was defined as the dose of peg-rhAgr1 that depletes arginine level to <8 μM throughout the treatment period. Optimal Biological Dose (OBD), which was defined as the lowest dose of peg-rhArg1 to achieve sustained ADD.

### Objectives

The primary objectives of this study were the safety, tolerability and in establishing the safety dose of peg-rhArg1 in the future clinical trials of advanced HCC patients. Secondary objectives included characterization of PK/PD and evaluation of treatment efficacy per RECIST 1.0 guideline [16].

### Disease evaluation

A full history and complete physical examination, including performance status were performed at every clinical visit. Disease assessment was performed by CT scan at baseline, week 11 and week 21. Response was independently reviewed by a designated radiologist at the Hong Kong Sanatorium and Hospital throughout the study and classified according to RECIST 1.0 criteria [16]. Patients who completed more than 8 weeks of the study were considered as evaluable for response. Weekly peg-rhArg1 administration continued (without dose escalation) for patients whose disease assessments showed either stable disease (SD) or response.

### Safety monitoring

Safety assessments consisted of monitoring and recording all the adverse and serious adverse events (AEs). Apart from monitoring of vital signs, regular collection of urine, hematology and blood chemistry of the enrolled subjects were performed. Patients who received at least one weekly dose of peg-rhArg1 were defined as Intent-to-treat population and considered evaluable for safety. AEs were evaluated according to National Cancer Institute Criteria (NCIC) version 3.0 [17].

### Statistical analysis

Survival analysis was computed by the Kaplan-Meier method. Progression-free survival (PFS) was calculated from the date of commencement of study drugs to the date of documented progression or death. OS was calculated from the date of commencement of study drugs to the date of death or last follow-up. The analysis was performed on intent-to-treat basis. All statistical analysis was performed by Statistical Package for Social Science version 13.0 for Windows (SPSS Inc., Chicago, IL USA).

## Results

### Demographics

Between May 2008 and July 2009, 15 patients were recruited in the study at weekly doses of 500 U/kg (3 patients), 1000 U/kg (3 patients), 1600 U/kg (3 patients) and 2500 U/kg (6 patients). Table 2 shows the demographics of all the recruited subjects. The median age was 57 years, and 12 patients (80%) were male. All except 2 were hepatitis B carriers; 2 received antiviral treatment for hepatitis B during peg-rhAgr1 treatment. Eleven (73.3%) patients were in Child-Pugh A cirrhosis, while the remaining four (26.7%) were in Child-Pugh B cirrhosis. All the recruited patients had advanced HCC that belonged to BCLC stage C (100%). Table 3 shows the details of the prior treatments in the enrolled patients. Ten (67%) patients had received prior treatments for HCC, while the remaining 5 patients were treatment naïve. Notably, seven (47%) patients were heavily pretreated with various chemotherapy regimens and anti-VEGF therapies.

**Table 2:** Demographics, baseline characteristic and prior treatment of 15 patients administrated with Peg-rhArg1

	No. of Patients	Percentage (%)
Characteristics		
Sex		
Male	12	80
Female	3	20
Age (years)		
Median	57	
Range	33–74	
Karnofsky Performance Scale		
100	6	40
90	7	46.7
80	2	13.3
Hepatitis Serology		
Hep Bs Ag positive	13	86.7
Child-Pugh Status		
A	11	73.3
B	4	26.7
Alpha-fetal Protein (AFP)		
<=400	5	33.3
>400	10	66.7
Disease stage at the time of study entry		
AJCC Staging		
IIIA	4	26.7
IIIC	3	20
IV	8	53.3
BCLC Staging		
C	15	100
Distant Metastases		
Lung	6	40
Bone	1	6.7
Adrenal	2	13.3
Brain	1	6.7
Invasion of Major Vessels		
Portal vein invasion	5	33.3
Prior Treatment		
Surgical treatment		
Liver resection	5	33.3
Laparotomy	1	6.7
Local ablative procedures		
TACE	6	40
RFA	1	6.7
Systemic therapy	7	46.7

*AJCC* American Joint Committee on Cancer staging; *BCLC* Barcelona Clinic Liver Cancer staging; *Hep Bs Ag* Hepatitis B surface antigen; *TACE* Transarterial chemo-embolization; *RFA* Radiofrequency ablation

**Table 3:** Summary of prior treatments in enrolled subjects

Subject Number	Prior Treatment Summary
101	Right Hepatectomy
102	TACE (Cisplatin) : 3X
201	Liver Resection;
	Radiofrequency Ablation;
	Doxorubicin: 6 cycles;
	TACE (Cisplatin): 2X;
	Anti-VEGF: 6 cycles
202	Right Hepatectomy;
	Doxorubicin: 6 cycles;
	Anti-VEGF: 6 cycles
203	Sorafenib: 8 cycles;
	Capecitabine: 8 cycles;
	Oxaliplatin: 8 cycles;
	TACE (Cisplatin): 4X
301	TACE (Cisplatin): 4X;
	Anti-VEGF: 9 cycles
302	Anti-VEGF: 4 cycles
403	Hepatectomy;
	Anti-VEGF: 2 cycles
	TACE (Cisplatin): 8X
405	Doxorubicin: 3 cycles;
	Anti-VEGF: 14 cycles;
	TACE (Cisplatin) : 8X
406	Hepatectomy only

*TACE* Transarterial chemo-embolization; *Anti-VEGF* Anti vascular endothelial growth factor

### Safety and tolerability

The median treatment exposure was 10 weeks (range, 2–23 weeks). Half of the patients had 6 to 12 weeks treatment exposure. Seven patients did not complete the 8 weeks of study drug. One out of three patients experienced possible DLT, in cohort 4 (2500 U/kg) with grade 4 bilirubin elevation in the first month of treatment. An additional 3 patients were recruited into this cohort as per protocol requirement. No further DLT was reported in these additional 3 patients. Therefore, the MTD was defined as 2500 U/kg.

In total, 9 possible drug-related AEs were recorded (Table 4). Most peg-rhArg1-related toxicities were grade 1 or 2, which resolved after discontinuation of peg-rhArg1. No treatment-related deaths were reported.

**Table 4:** Summary of possible treatment related Adverse Events (AEs)

	Total No. of AEs			
	Grade 1 (%)	Grade 2 (%)	Grade 3 (%)	Grade 4 (%)
Abdomen Pain	1 (6.7)			
Diarrhea	2 (13.3)			
Nausea		1 (6.7)		
Elevated ALT			1 (6.7)	
Elevated AST			1 (6.7)	
Elevated Bilirubin			1 (6.7)	1 (6.7)
Elevated GGT			1 (6.7)	

All AEs were scored according to the National Cancer institute (NCI) Common Terminology Criteria, Version 3, June 10, 2003

*ALT* Alanine Transaminase; *AST* Asparate Transaminase; *GGT* Gamma-glutamyl transferase

### Pharmacokinetics

**Fig. 2:**
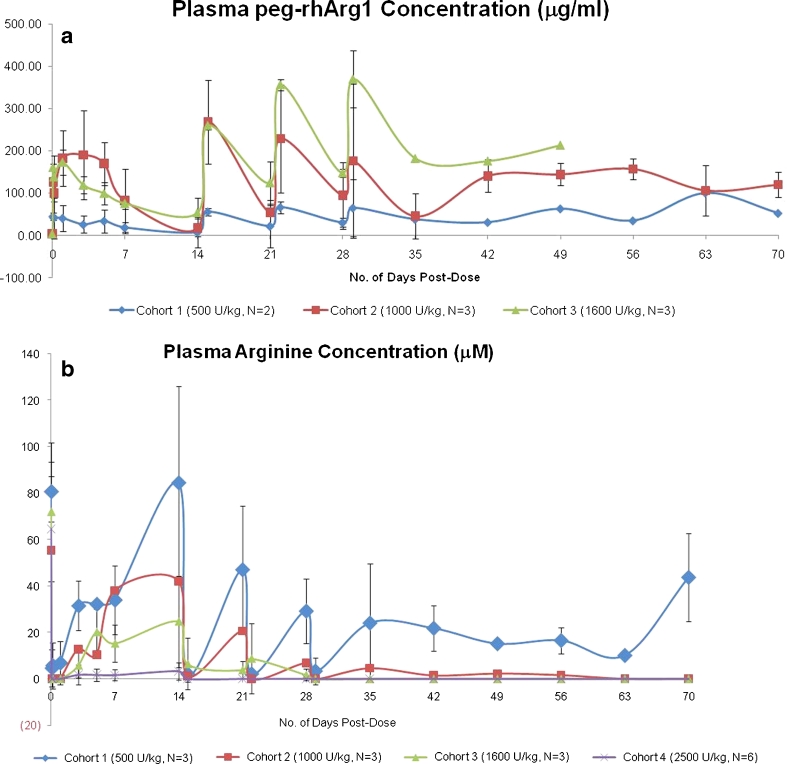
**a**. Plasma Peg-rhArg1 level after single and multiple doses of Peg-rhArg1 in HCC patients **b**. Plasma arginine concentration after single and multiple dosing of Peg-rhArg1 in HCC patients

Figure 2a shows the pharmacokinetics of peg-rhArg1 in cohort 1-3. Following the first dose of peg-rhArg1, the mean C_max_ at 500, 1000, 1600U/kg were 78.8 (86.5%), 212.0 (35.2%), and 171.3 (17.3%) μg/ml, respectively. The mean peak-through ratios were 4.23 in cohort 2, and ranged from 2 to 3 for other 2 cohorts. The mean AUC_∞_ was about 8.94 (28.2%) mg*h/ml for cohort 1 (500U/kg). Cohort 2 (1000U/kg) and 3 (1600U/kg) had similar mean AUC_∞_, which were 34.67 (58.4%) and 33.36 (20.5%) mg*h/ml respectively.

### Pharmacodynamics

Peg-rhArg1 was efficacious in inducing arginine depletion in a dose-dependent manner. Figure 2b shows the relationship between plasma arginine levels after peg-rhArg1 dosing. The time taken to achieve undetectable plasma of arginine was 2 h for all patients following a single dose. The 2500U/kg dose level in cohort 4 had the greatest arginine depletion and the highest median duration of the ADD effect (166 h). In cohort 3 (1600U/kg), the effective ADD was achieved after the second weekly dose of peg-rhArg1 and ADD was maintained throughout the treatment period. The lowest dose of peg-rhAgr1 that achieved ADD was 1600U/kg in cohort 3 and this dose was therefore deemed the OBD.

### Recommended safe dose of Peg-rhArg1

The study was terminated on completion of all the 6 cases in cohort 4 since both the MTD (2500 U/kg) and OBD (1600U/kg) were established. The final recommended safe dose for future clinical trials was chosen as 1600U/kg instead of 2500 U/kg based on the premise that it was the lower tolerated dose of peg-rhArg1 to achieve adequate arginine depletion.

### Treatment efficacy and survival analysis

**Fig. 3:**
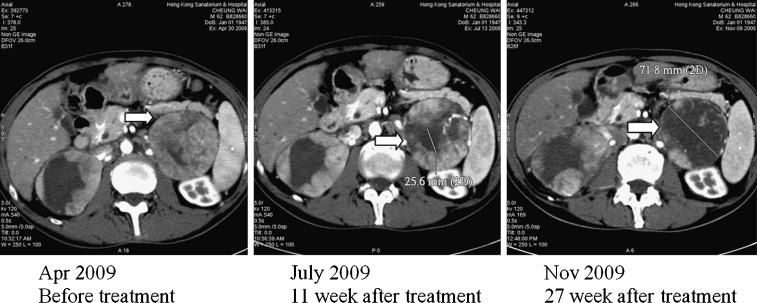
Computerized Tomographic (CT) Scan of HCC Patient: Serial CT scan images of liver tumor before treatment, 11 weeks after treatment showing the increase of necrosis with diameter of 25.6 mm, and 27 weeks after treatment showing necrosis with diameter of 71.8 mm, respectively

Of the 15 patients recruited, 7 patients were terminated before formal radiological assessments for efficacy due to the following reasons: 2 died of liver failure from advanced cirrhosis, 3 patients died of rapid progressive diseases, 1 had prolonged elevation of bilirubin (DLT) and 1 withdrew voluntarily from treatment. Overall, 4 (26.7%) patients achieved SD and was observed at all 4 dose levels, one from each cohort. Notably, marked tumor necrosis was observed in two cases without actual change in tumor dimensions on CT (Fig. 3). The median baseline alpha-fetal protein (AFP) level was 954 μg/L (range, 4-267000). Among all the 15 enrolled patients, six patients (40%) showed a decline in AFP compared with baseline during the study.

**Fig. 4:**
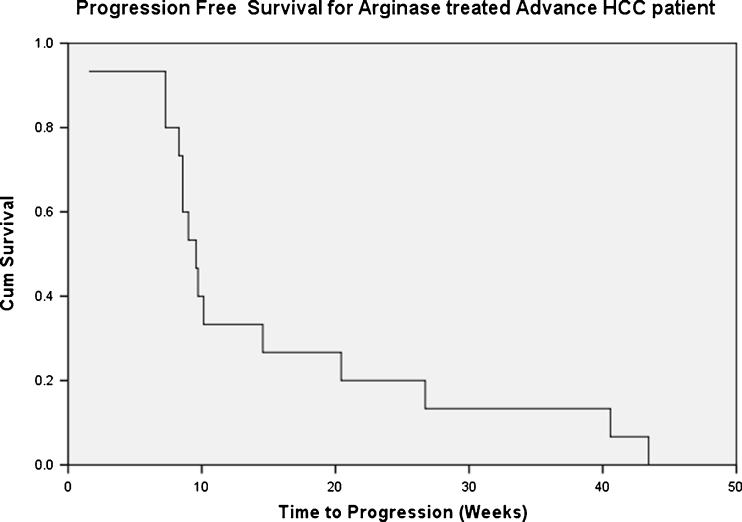
Kaplan-Meier Progression-free Survival (PFS) of enrolled patients

The median PFS and OS for the entire study population of 15 patients were 2.8 months (95% CI: 0.2–3.0 months) (Fig. 4) and 5.1 months (95% CI: 0.2–14.8 months) respectively. The patients with Child-Pugh A cirrhosis had significantly better median OS than the Child-Pugh B cirrhotic patients (8.5 versus 1.8 months, *p* < 0.008). Two patients receiving peg-rhArg1 at 1000 U/kg and 1600 U/kg remained SD for >6 months.

## Conclusion

This is the first human clinical study using peg-rhArg1 in the treatment of advanced human malignancy. We have demonstrated that arginine depletion in humans can be achieved safely with peg-rhAgr1 in a dose-response manner. There was no treatment-related death and all the AEs, except for the one possible case of DLT in cohort 4, were mild grade 1 and 2 non-hematologic toxicities, such as nausea and abdominal discomfort, which resolved spontaneously with symptomatic treatment. Thus, the primary objectives of the study were met and the recommended weekly dose for future development of Peg-rhAgr1 was chosen as 1600U/kg.

As in any drug trials involving patients with severely compromised liver functions, there is always a problem in attributing the exact cause of dysfunction, whether due to the test drug or progression of underlying liver disease, i.e. cirrhosis, hepatitis or malignancy, often all three. Overall, only 2 of the 15 patients in the entire study had possible severe drug-related liver dysfunction, one in cohort 1 and the other in cohort 4. The first patient had grade 3 elevation of ALT/AST after the first injection and was taken off the study. Before he went into hepatic encephalopathy with liver failure, a CT scan confirmed progression of the liver tumour, from which he died after 2 months off treatment. The second case had grade 3 followed by 4 hyperbilirubinemia after a single injection of peg-rhArg1. Since the patient had no overt clinical signs of disease progression, this hyperbilibrubinemia was deemed as DLT. Although great care was taken to recruit patients with adequate life-expectancy into the study, the poor outcomes of these patients early on in their treatment illustrate how unpredictable the clinical course of heavily pre-treated HCC patients with advanced disease can be. In particular, nearly half of the recruited patients were heavily pre-treated with various prior system therapies, which might account for the rapid deterioration of the recruited patients in the study. As per protocol requirement, the additional 3 cases were recruited into cohort 4, and it was reassuring that no further liver DLT was observed. The study was terminated on completion of all the 6 cases in cohort 4 since the MTD and OBD—the two important objective parameters the authors aimed to establish—had already been found.

In terms of toxicity profiles, peg-rhAgr1 does have an advantage over both ADI and sorafenib. When compared with ADI, peg-rhAgr1 releases urea as a side product, which is much less toxic than the ammonia that is released by ADI treatment. This is important since in patients with compromised liver function, as in most HCC cases, the extra ammonia may make the patients more susceptible to develop HE. Indeed, the most common AEs attributed to ADI include transient and reversible HE. Other toxicities reported in ADI included skin and injection site necrosis and electrolyte disturbance, which were not seen in our study. Being a *Mycoplasma* product, ADI is a xeonbiotic immunogenic substance, despite pegylation. It has been reported that neutralizing antibodies to ADI developed ~50 days after initiation of treatment [11]. Peg-rhAgr1, on the other hand, is a recombinant human hepatic enzyme and in our preclinical and clinical studies (data not shown); no evidence of immunogenicity has been found to date. This has important therapeutic implication since the neutralizing antibodies would render the drug ineffective as an arginine-depleting agent. It was also reported in the ADI studies that arginine levels would rise in the second month of treatment, coinciding with the development of neutralizing antibodies to ADI. On the other hand, neutralizing antibodies to Peg-rhAgr1 have not been detected thus far in the present study and it is reassuring that arginine levels remained at lower levels throughout the entire study as long as treatment continued. More importantly, the AEs profile of peg-rhAgr1 also compares favourably with that of sorafenib, which has large numbers of grade III and IV toxicities in terms of hand-foot-skin reaction, hypertension and rash. Again, none of these were seen in this study.

Enzymatic depletion or deprivation therapy, whether with peg-rhAgr1 or ADI, has now emerged as a new platform of anti-cancer treatment in a number of human malignancies, such as HCC and melanoma [18]. In vitro and in vivo xenograft studies suggest that this mode of treatment is efficacious also in prostatic and renal cell cancers, in which ASS and arginosuccinate lyase (ASL) were highly repressed or absent in an MD Anderson series [19]. Pegylated human arginase has recently been shown in one US study to induce remission in acute T cell leukemia [20]. The present study serves to prove the concept that arginine depletion is really a promising approach in treating advanced HCC patients. Thus, further evaluation of peg-rhArg1 in future phase-II trials of advanced HCC is warranted.

Peg-rhAgr1 might have demonstrated early activity in the group of heavily pre-treated advanced HCC patients. Of the 8 cases completed 8 weeks of treatment and beyond, 4 had durable SD lasting for 3 m+, 6 m+, 6 m+ and 3 m+, respectively with drop in AFP level. One patient had minor shrinkage of the target lung lesions that did not qualify as a partial response by the RECIST 1.0 criteria. As experienced by other clinical investigators in the field of HCC, assessing CT response to treatment in HCC is notoriously difficult and tumour response may result in tumour necrosis instead of shrinkage (Fig. 2) [21]. Notably, at the time when this study was conducted, RECIST 1.0 criteria was the standard for assessing tumor response [22]. As yet, there is no consensus in the HCC community about which new radiological criteria, such as EASL [13] or modified RECIST [21] would be a better method to assess response in HCC patients receiving targeted therapy or biological therapy alone. Unfortunately, 7 enrolled patients were not evaluable for treatment response and most of these patients discontinued the therapy early mainly due to either rapid disease progression or various complications from underlying cirrhosis. This indeed reflects the challenges in conducting clinical trials in this refractory patient population with aggressive tumour biology and poor liver reserve.

In conclusion, peg-rhArg1 has a manageable safety profile and is potentially a superior arginine depleting agent than ADI in the treatment of human malignancies due to its low toxicity profile and sustainable arginine depletion. The optimal therapeutic dose of peg-rhArgI is 1600U/kg in humans for future studies in advanced HCC patients.
